# 3D multi-view convolutional neural networks for lung nodule classification

**DOI:** 10.1371/journal.pone.0188290

**Published:** 2017-11-16

**Authors:** Guixia Kang, Kui Liu, Beibei Hou, Ningbo Zhang

**Affiliations:** 1 School of Information and Communication Engineering, Beijing University of Posts and Telecommunications, Beijing, China; 2 Beijing Ciji Network Technology Co., Ltd., Beijing, China; Southwest University, CHINA

## Abstract

The 3D convolutional neural network (CNN) is able to make full use of the spatial 3D context information of lung nodules, and the multi-view strategy has been shown to be useful for improving the performance of 2D CNN in classifying lung nodules. In this paper, we explore the classification of lung nodules using the 3D multi-view convolutional neural networks (MV-CNN) with both chain architecture and directed acyclic graph architecture, including 3D Inception and 3D Inception-ResNet. All networks employ the multi-view-one-network strategy. We conduct a binary classification (benign and malignant) and a ternary classification (benign, primary malignant and metastatic malignant) on Computed Tomography (CT) images from Lung Image Database Consortium and Image Database Resource Initiative database (LIDC-IDRI). All results are obtained via 10-fold cross validation. As regards the MV-CNN with chain architecture, results show that the performance of 3D MV-CNN surpasses that of 2D MV-CNN by a significant margin. Finally, a 3D Inception network achieved an error rate of 4.59% for the binary classification and 7.70% for the ternary classification, both of which represent superior results for the corresponding task. We compare the multi-view-one-network strategy with the one-view-one-network strategy. The results reveal that the multi-view-one-network strategy can achieve a lower error rate than the one-view-one-network strategy.

## Introduction

Lung cancer is the most frequently diagnosed cancer and was the most leading cause of cancer death among males in 2012 [1]. Clearly, lung cancer has become a major threat to human life. However, people with early stage lung cancer do not present any clinical symptoms. Patients only begin presenting symptoms once the lung cancer has sufficiently advanced. Therefore, early detection is crucial for lung cancer survivability, and can improve the effectiveness of treatment and increase the patient’s chance of survival.

Low-dose computed tomography (CT) is an effective method for identifying lung cancer early. However, radiologists must carefully examine each image from amongst a very large number of CT images, greatly increasing the burden of labor on radiologists. On the other hand, radiologists tend to be subjective when using CT images for the diagnosis of lung disease, often leading to inconsistent results from the same radiologist at different times or from different radiologists examining the same CT image. To alleviate these diagnostic challenges, computer aided diagnosis systems, which use an automated image classification technique, can be used to help radiologists in terms of both their accuracy and speed.

Most traditional methods for the automated classification of nodules do not work in an end-to-end manner: first, they extract features using predefined filters, such as descriptors of histograms of oriented gradients [2], and wavelet feature descriptors [3], or they extract hand-crafted features, such as those related to geometry [4, 5], appearance [6] or texture [7].

An alternative to identification through these predefined features is by using feature learning methods to learn high-level representations directly from the training data. Convolutional neural networks (CNN), as a fast, scalable, and end-to-end learning framework, drastically advanced the landscape of computer vision, such as in image classification [8], object detection [9], semantic segmentation [10], and action recognition [11] tasks, etc. However, the convolutions in these CNN models are all two-dimensional (2D).

Recently, the architecture of CNN has undergone some improvements. LeCun et al. [12] designed LeNet-5, in which the convolutional layer and the pooling layers were alternately connected. This model is widely used in the United States to identify numbers on hand-written checks. Krizhevsky et al. [13] constructed a large-scale CNN on two GPUs, which definitively won the ILSVRC-2012 competition. Lin et al. [14] added 1×1 convolutional layers to their network to increase representational power, which was heavily used in GoogLeNet [8, 15, 16]. The Visual Geometry Group (VGG) [17] encouraged model designers to use small convolutions in order to build deeper networks. Later, Google designed several versions of CNN-based on Inception architecture. The first version of the Inception architecture (Inception v1) made use of different kernel sizes in the same convolutional layer [8]. Batch Normalization (BN) was introduced in Inception v2 [18]. In Inception v3, larger convolutions were designed to be divided into multiple small convolutions, while *n* × *n* convolutions were designed to be divided into 1 × *n* and *n* × 1 convolutions [15]. Almost simultaneously, deep residual network was proposed by He et al. [19] and achieved a top-five error rate of 3.57% in the ILSVRC-2015 classification task. Later, Google introduced residual connections in the Inception network, and proposed Inception-resNet-v1 and Inception-resNet-v2 [16], in which the top-5 error rate for the Imagenet classification challenge was reduced to 3%, with an ensemble of three residual and one Inception-v4.

Motivated by the success of CNN in the field of image recognition, there have been efforts made to apply the technique to medical diagnosis, especially nodule classification in CT. Kumar et al. used auto-encoders [20] and CNN [21] to classify lung nodules as either malignant or benign, reaching a best accuracy of 77.52%. Shafiee et al. [22] leveraged stochastic sequencers consisting of three stochastically-formed convolutional layers and obtained an accuracy of 84.49%, a sensitivity of 91.07%, and a specificity of 75.98%. Kim et al. [23] used a stacked denoising auto-encoder with 3 hidden layers to extract features from collected CT scans. They combined these features with 96 raw hand-crafted imaging features and fed them to a SVM classifier. The results showed that this method was more effective than the conventional methods that used only the original raw hand-crafted features. Shen et al. [24] proposed multi-scale convolutional neural networks that could capture nodule heterogeneity by extracting discriminative features from alternatingly stacked layers. They [25] then modified their model and presented a multi-crop convolutional neural network that was able to automatically extract salient nodule information by employing a novel multi-crop pooling strategy that crops different regions from convolutional feature maps and applies max-pooling at varying times.

The use of 3D CNNs in the field of medical imaging applications is still in its infancy. Dou et al. [26] used multi-level 3D CNNs with the aim of reducing false positive in lung nodule detection. The proposed algorithm achieved the highest CPM score in LUNA16. There are also other variants of 3D CNNs for medical imaging applications. Dou et al. also used a 3D CNN combined with conditional random fields to segment a liver from CT images. They evaluated the model’s perforemance on the public dataset MICCAI-Sliver07 and obtained a state-of-the-art effect [27]. They also used a 3D fully convolutional network (FCN) to retrieve cerebral micro-bleeds (CMBs) candidates and then applied a 3D CNN to distinguish CMBs from mimics, yielding a sensitivity of 93.16% in a data set with 320 volumetric magnetic resonance scans [28]. Çiçek et al. [29] proposed a 3D u-net by replacing all 2D operations in u-net with their 3D counterparts for volumetric segmentation. Kamnitsas et al. [30] proposed a dual way 3D CNN combined with 3D full-chain Conditional Random Fields employed to reduce false positive. The pipeline was evaluated by its performance on 3 challenging tasks of lesion segmentation in multi-channel Magnetic Resonance Imaging data with traumatic brain injury, brain tumors, and ischemic stroke.

Thoracic CT produces a volume of slices that can be manipulated to demonstrate various volumetric representations of bodily structures in the lung. 2D convolution ignores the third spatial dimension, meaning it is unable to make full use of the 3D context information, 3D CNN can, obviously, make up for this. We investigate empirically the challenge of classifying lung nodules captured by computed tomography (CT) in an end-to-end manner using the 3D multi-view convolutional neural networks (MV-CNN), and conduct a binary classification (benign and malignant) and a ternary classification (benign and malignant primary and metastatic malignant) on CT images from the Lung Image Database Consortium image collection (LIDC-IDRI). Our main contributions can be summarized as follows:

1. We use 3D CNN for automatic classification of lung nodules. Compared with the 2D model, 3D CNNs can encode richer spatial information to extract more distinguishable representations.
2. Multi-view patches are used in our models. However, we use the multi-view-one-network strategy that differs from the one-view-one-network strategy used in paper [26]. The results show that our strategy can achieve a lower error rate than the one-view-one-network strategy while using fewer parameters. Note that, while the model employed in paper [31] used a similar strategy, they employed only 2D CNN, while we used 3D CNN for this paper.
3. To the best of our knowledge, this is the first study to use 3D variants of Inception and Inception-ResNet to classify lung nodules. In the latter part of this paper, the 3D variants of Inception and Inception-ResNet will be writtern as “3D Inception” and “3D Inception-ResNet” respectively, for brevity’s sake.
4. Our model achieved better results than other works related to the classifications on CT images from LIDC dataset.

## Materials and methods

### Data

Data from the LIDC-IDRI database [32] is used in our experiment. It consists of 1018 lung cancer screening thoracic CT cases with marked-up annotated lesions. They are all annotated by 4 experienced thoracic radiologists. The annotated lesions are divided into three categories: “nodule> = 3 mm”, “nodule<3 mm” and “non-nodule> = 3 mm”. In order to validate the training and evaluation protocols of our classification system, we chose to use the ratings from diagnostic data, which is the only available way to judge the certainty of malignancy. However, diagnostic data is available for 157 patients only. In order to extract information for these patients, we chose to use the LIDC Image Toolbox [33] developed by Thomas Lampert. However, the toolbox was unable to extract information for some patients, and, therefore, these patients were removed from our experiment, leaving only 96 final patient participants. The diagnostic result was obtained at two levels: i) patient level and ii) nodule level. At each level, the lesions in the lung were marked as either:

0—Unknown
1—benign or non-malignant disease
2—malignant, primary lung cancer
3—malignant metastatic

### 3D CNNs

In general, a CNN consists of convolutional, pooling and fully-connected layers to extract multi-level learnable representations. They are learned jointly, in an end-to-end manner, to solve a particular task. Unlike the conventional CNN, each channel in a 3D CNN is actually a 3D feature volume, rather than a 2D feature map. The convolutions and poolings of 3D CNN are operated in a cubic manner. We introduce some of the basic components of 3D CNN in the following paragraphs.

#### 3D convolutional layer

We use ***y*** = *conv*(***x***, ***w***) to denote the convolutional function operated by the 3D convolutional layer, where ***x*** represents the original data or feature maps that the convolutional function operate on, ***w*** denotes the filters and ***y*** denotes the output of the convolutional layer. The input ***x*** has *Z* × *M* × *N* × *K* × *S* dimensions, where *Z* denotes the length (the number of slices in z axis) of the map, *M* and *N* represent, respectively, the height and width of the map, *K* is the number of channels, and *S* is the batch size. Note that each filter has a dimension *Z*_*f*_ × *M*_*f*_ × *N*_*f*_ × *K* × *K*′, where *Z*_*f*_, *M*_*f*_ and *N*_*f*_ are the length, height and width of the filters, respectively. It operates on map ***x*** with *K* channels, generating map ***y*** with *K*′ channels as follows

$$y_{l^{\prime }i^{\prime }j^{\prime }k^{\prime }s}=\sum_{lijk}w_{lijkk^{\prime }}x_{l+l^{\prime },i+i^{\prime },j+j^{\prime },k,s}.$$ (1)

#### Activation function

The activation function, which is applied to each component of a feature map, introduces non-linearity in a CNN. We use the *Rectified Linear Unit* (*ReLU*) as the activation function in this paper. It works as follows:

$$y_{lijks}=max\{0,x_{lijks}\},$$ (2)

using ***y*** = *ReLU*(***x***) to represent it.

#### 3D pooling layer

The pooling layer is another important operator in a CNN. A pooling operator runs on individual feature channels, coalescing nearby feature values into one via the application of a suitable operator. Common choices for this include max-pooling or average-pooling. We prefer to use max-pooling, just like several other researchers [13, 17], which is defined as

$$y_{lijks}=max\{x_{l^{\prime }i^{\prime }j^{\prime }ks}:l\leq l^{\prime }<l+p_{\text{1}},i\leq i^{\prime }<i+p_{\text{2}},\;\;j\leq j^{\prime }<j+p_{\text{3}}\},$$ (3)

where *p*_*i*_ denotes the pooling size. ***y*** = *MaxP*(***x***) is used to represent it. In some networks, we use 3D averaging pooling defined as

$$y_{lijks}=\frac{1}{p_{1}\times p_{2}\times p_{3}}\sum_{\begin{array}{l} l\leq l^{\prime }<l+p_{\text{1}},\\ i\leq i^{\prime }<i+p_{\text{2}},\;\;\\ j\leq j^{\prime }<j+p_{\text{3}} \end{array}}^{}x_{l^{\prime }i^{\prime }j^{\prime }ks}.$$ (4)

As can be seen from the above equations, the output of each layer is also a 5D tensor, where the meaning of each dimension is the same as the input ***x***.

#### Fully-connected layer

Each neuron in a fully-connected layer is connected with all neurons in adjacent layers. AlexNet [13] and VGG [17] still retain the fully connected layer, which greatly increases the number of network parameters. So, in recent years, as the depth of networks has grown, researchers have tended to abandon the fully connected layer and replace it with an average pooling layer. This method can simultaneously both reduce significantly the parameters of a network and maintain the generalization ability at the same time [14].

#### Dropout and spatial dropout

We applied dropout on the fully connected layer. Dropout is a strategy proposed by Hinton et al. [34] to relieve over-fitting for neural networks. Specifically, if the dropout rate of a layer is *r* (0 <*r* <1) and the number of parameters is *N*, then the model will only update randomly selected *Nr* parameters during the training phase, while all parameters are used in the inference phase. We applied spatial dropout [35] on the convolutional layer. For a given convolution feature tensor of size *n*_*f*_×*height*×*width*, the spatial dropout performed only *n*_*f*_ dropout trials and extended the dropout value across the entire feature map. Therefore, adjacent pixels in the dropped-out feature map are all either 0 (dropped-out) or active [35]. This breaks the entire feature map, not just a single neuron.

#### Global average pooling

The idea of global average pooling is to generate one feature map for each corresponding category of the classification in the last 1×1×1 convolutional layer. Instead of adding fully connected layers on top of the feature maps, it takes the average of each feature map and feeds the resulting vector directly into the softmax layer. GoogLeNet [8,15,16] also uses global average pooling to replace the fully connected layer. Lin et al.[14] think it not only effectively reduces the parameters, but also make feature maps that can be easily interpreted as category confidence maps, and the network is more robust for spatial translations of the input.

### CNN architecture

In conventional CNNs, such as LeNet and AlexNet, computational blocks form a simple chain; however, within more complex topologies, such as the residual net and GoogLeNet, blocks are interconnected to form a directed acyclic graph (DAG). In this paper, both 3D CNN with chain architecture and with DAG architecture are explored.

#### The multi-view strategy

**Fig 1:**
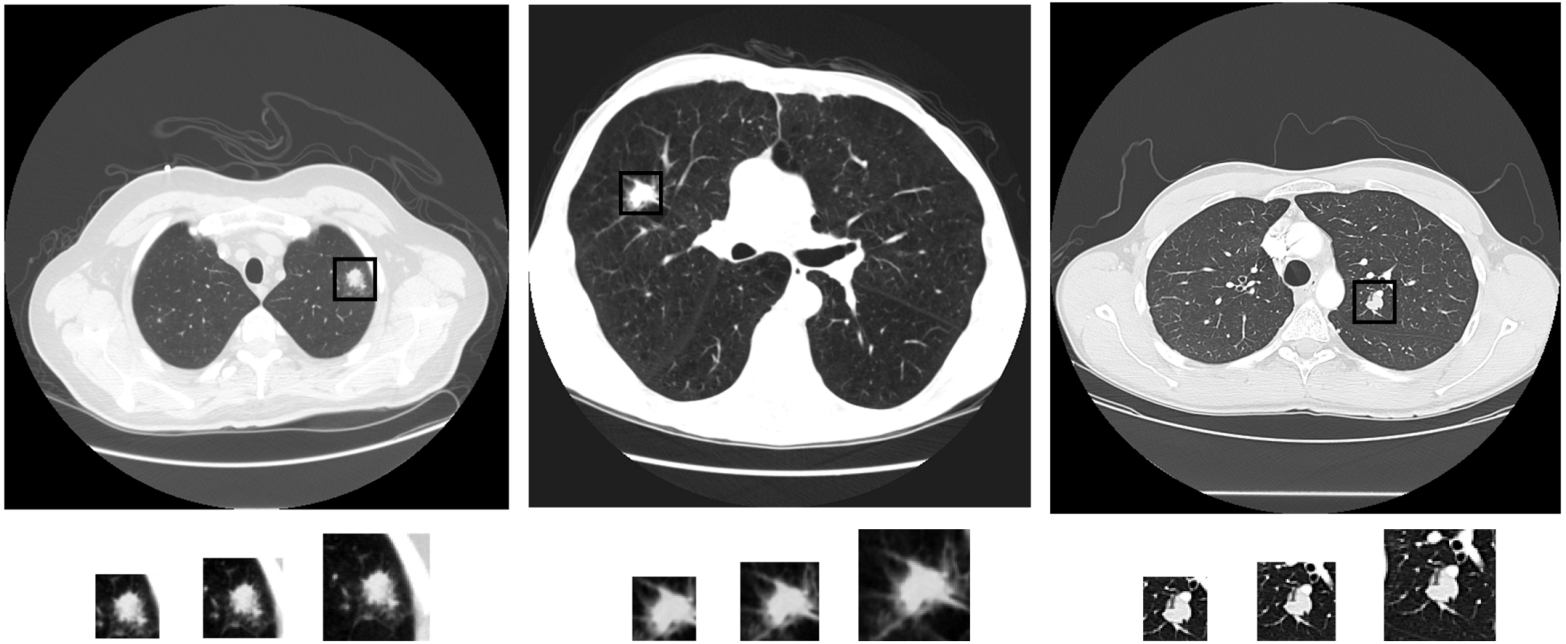
CT examples with lung nodules in different categories. They are benign (left), primary malignant (middle) and metastatic malignant (right), alone with 3 different view areas including 40×40, 50×50 and 60×60.

We use the multi-view strategy illustrated in Fig 1. Specifically, we first find the geometrical center of each nodule. Centered on these points, we crop patches in different sizes, offering different view areas. We resize them into the same size using spline interpolation before feeding them into different channels.

**Fig 2:**
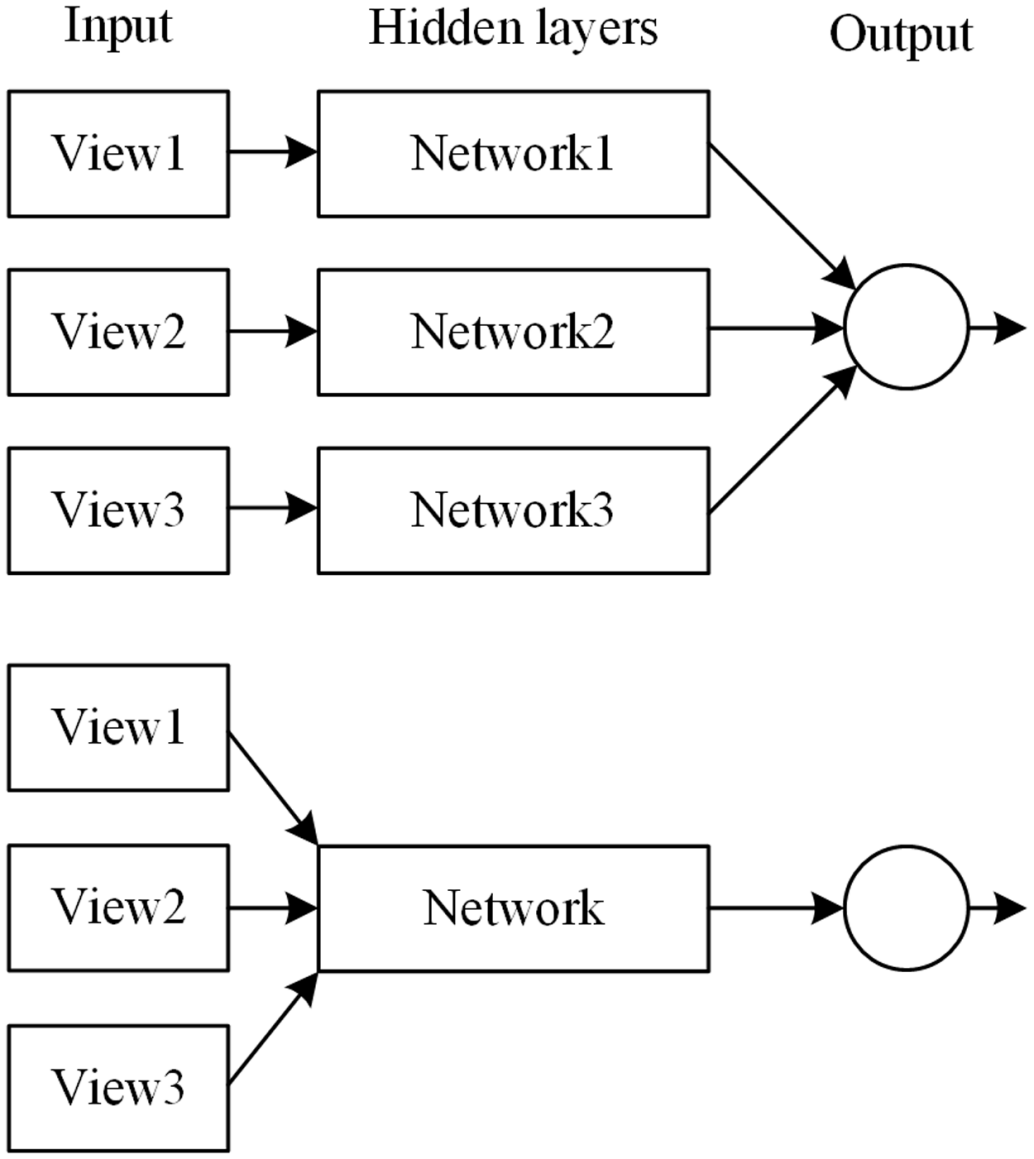
Two kinds of the multi-view strategy. The one-view-one-network strategy (top) employs a separate network for images from each view (cropping size), while the multi-view-one-network strategy (bottom) uses one network for all views.

The muti-view strategy has two methods of implementation, which, in this paper, we dub as the one-view-one-network strategy and the muti-view-one-network strategy in this paper. Fig 2 shows the difference between them. Dou et al. [26] used the former while we use the latter.

#### The 3D CNN with chain architecture

**Fig 3:**
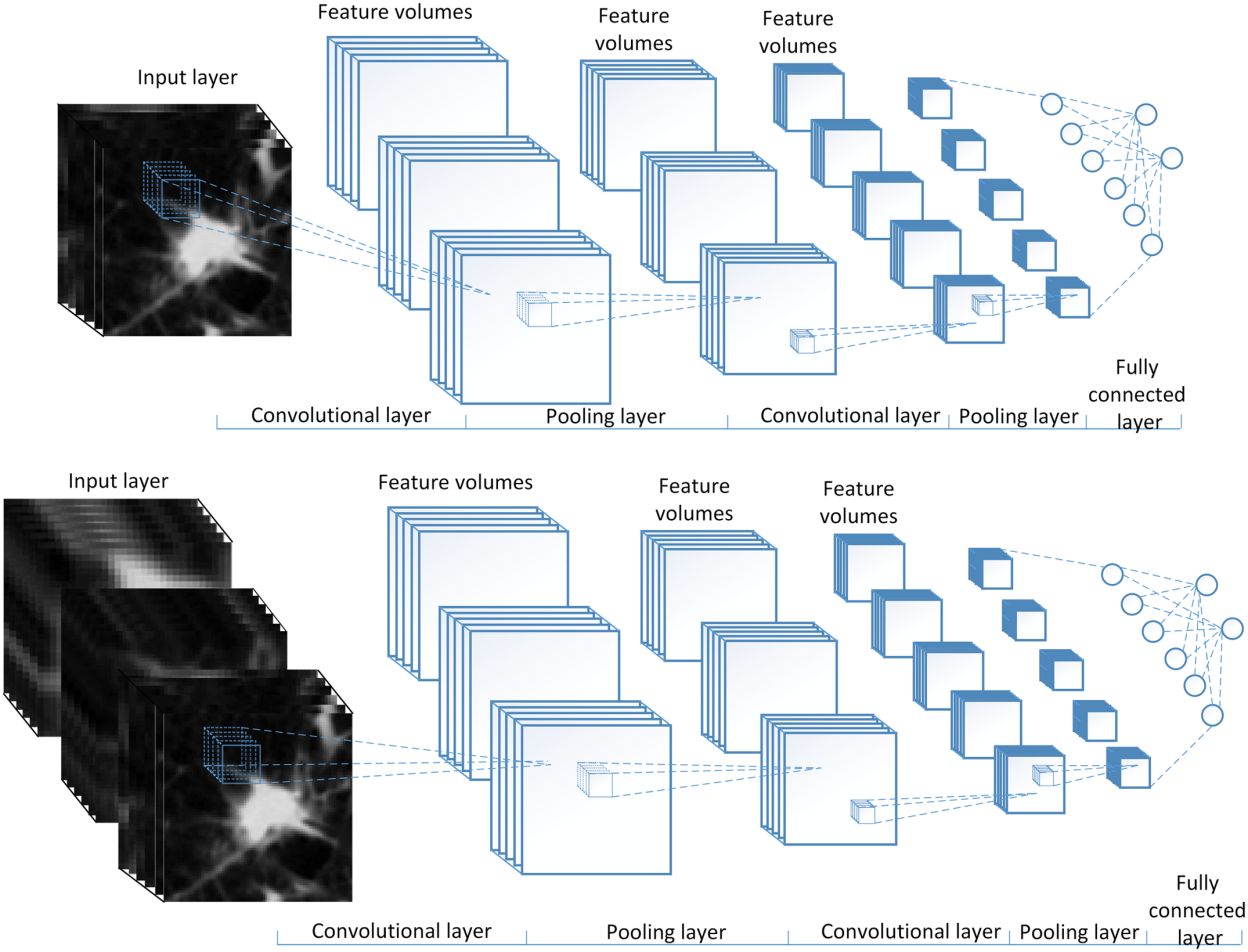
The architectures of the 3D single view CNN (SV-CNN) (top) and 3D MV-CNN (bottom).

In 3D CNN with chain architecture, the convolutional layer and the pooling layer are alternately connected. A channel of the input in 3D CNN is organized in a cubic manner. So, each channel of the hidden layer is actually a 3D feature volume. The architectures of 3D CNN with a single view and with multiple views are shown in Fig 3.

In order to observe and evaluate performance with regard to network configuration, we investigated different configurations, including the number of input channels and the depth of the network. The MV-CNN had *n* input channels and *m* convolutional layers, where *n* = {1,3}and *m* = {0,1,2,3}. We use

m•(MaxP(ReLU(conv(x,w))))+fc+softmax (5)

to describe the structure of the network, where *MaxP*, *ReLU* and *conv* are described in the previous section and *fc* denotes the fully connected layer. We show the filter size and the number of channels in each layer for different network architecture in Table 1. We think that a node in the fully connected layer is a channel, so the number “128” in the last column refers to the number of nodes in the fully connected layer.

**Table 1:** The configuration of the network with chain architecture.

Number	Architecture	Filter size	The number of channels
Softmax	s*oftmax*	-----	----
CNN1	*MaxP*(*ReLU*(*conv*(***x***,***w***)))+*fc*+s*oftmax*	3×5×5	20,128
CNN2	2•(*MaxP*(*ReLU*(*conv*(***x***,***w***))))+*fc*+s*oftmax*	3×5×5, 3×3×3	20,50,128
CNN3	3•(*MaxP*(*ReLU*(*conv*(***x***,***w***))))+*fc*+s*oftmax*	3×5×5,3×5×5,2×3×3	20,50,50,128

#### The 3D CNN with DAG architecture

**Fig 4:**
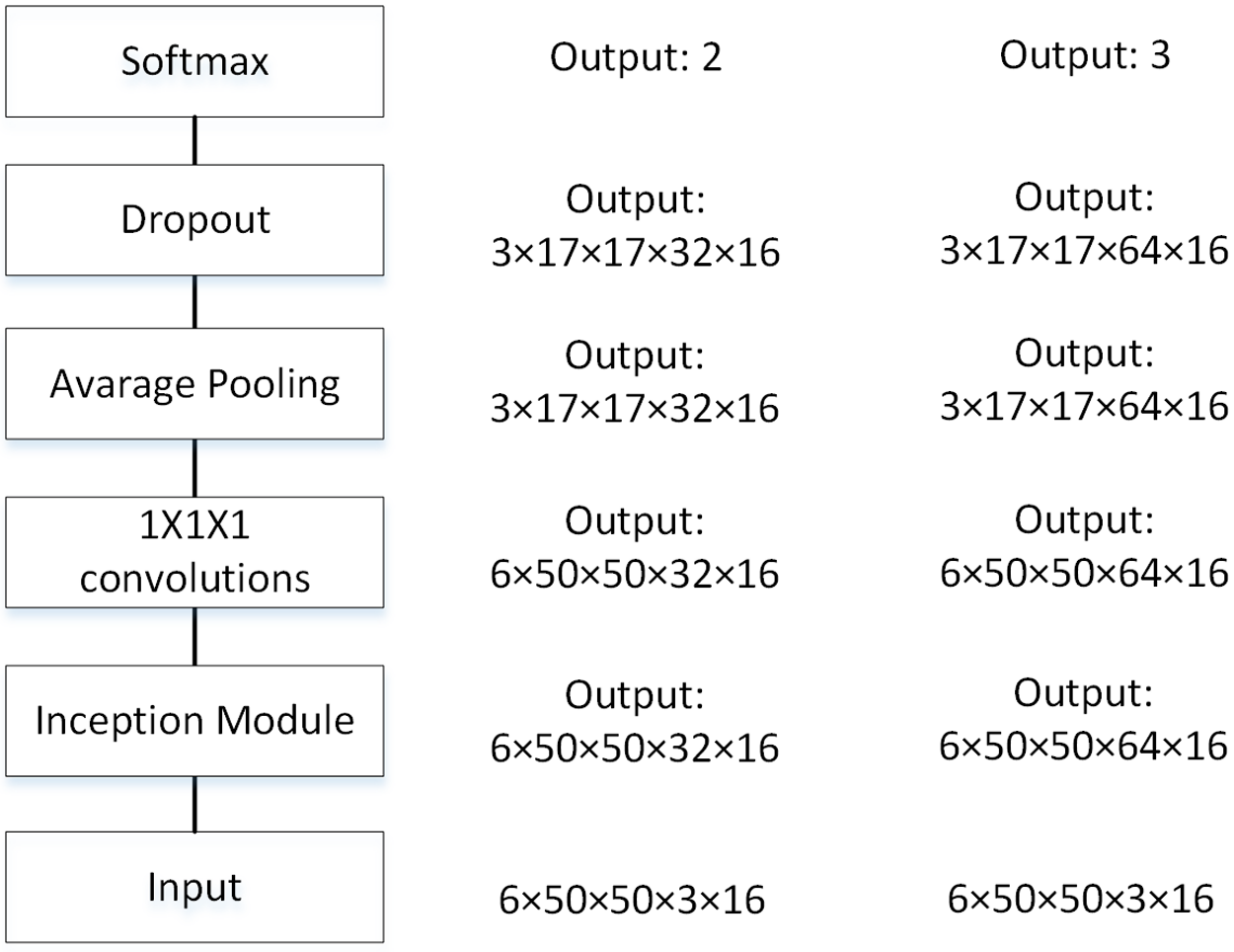
**The overall architecture of the 3D multi-view Inception network (left)**. This architecture applies to Inception1 and Inception2. The output sizes of each layer for the binary classification (middle) and the ternary classification (right) are different, which are also shown in this figure. Note that the output of each layer is a 5D tensor as described in the previous section. The details of the Inception module are shown in Fig 5.

**Fig 5:**
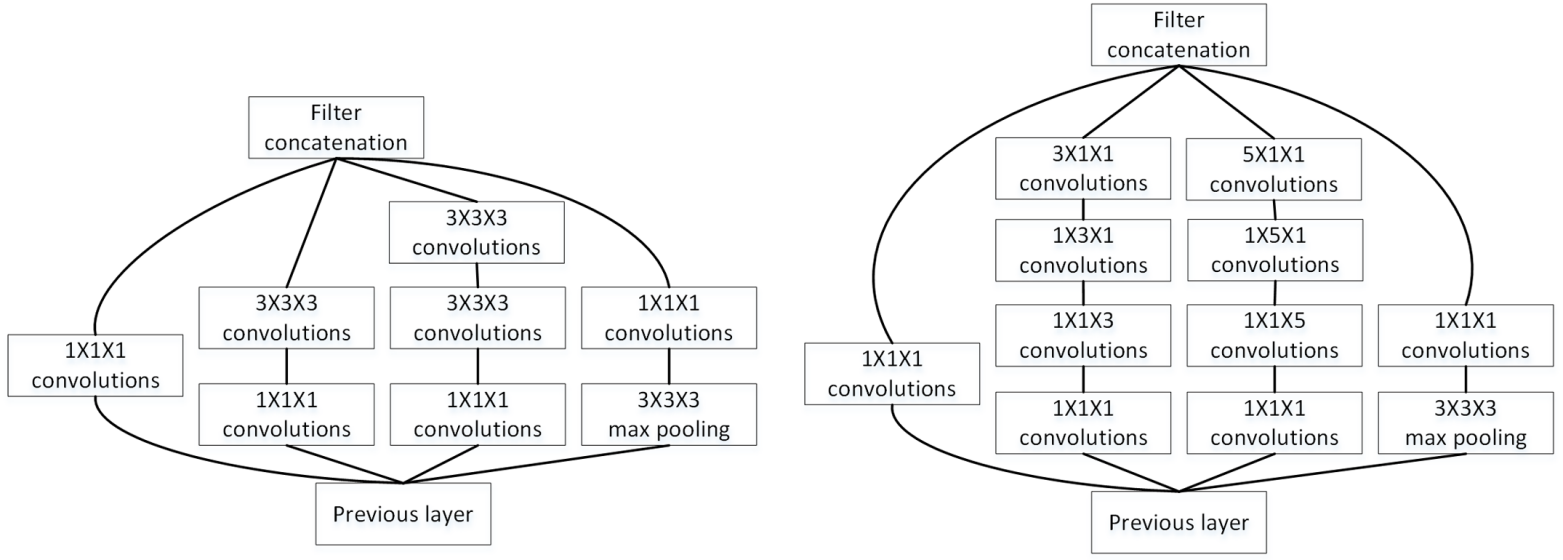
The detail of the Inception module in Fig 4: 3D Inception1 (left) and 3D Inception2 (right).

The 3D Inception model divides the network into multiple branches, each with a different pooling size. Fig 4 shows the overall architecture of the 3D Inception network. It should be noted that, inspired by Min Lin et al. [14] and Szegedy et al. [16], we removed the fully-connected layer in the Inception network. In fact, we wanted to imitate the global average pooling method, but we did not fully follow this method. We used the 1×1×1 convolutional layer and average pooling layer to replace the fully connected layer, but we used more channels and smaller pooling size. There was only one Inception module in the overall architecture. The detailed structures of this Inception module are shown in Fig 5. We employed two kinds of 3D Inception modules. The first made use of 1×1×1, 3×3×3 and 5×5×5 kernel sizes, while the second divided the *n*×*n*×*n* (*n* = 1, 3, 5) filter into 3 filters with kernel sizes of 1×1×*n*, 1×*n*×1 and *n*×1×1.

**Fig 6:**
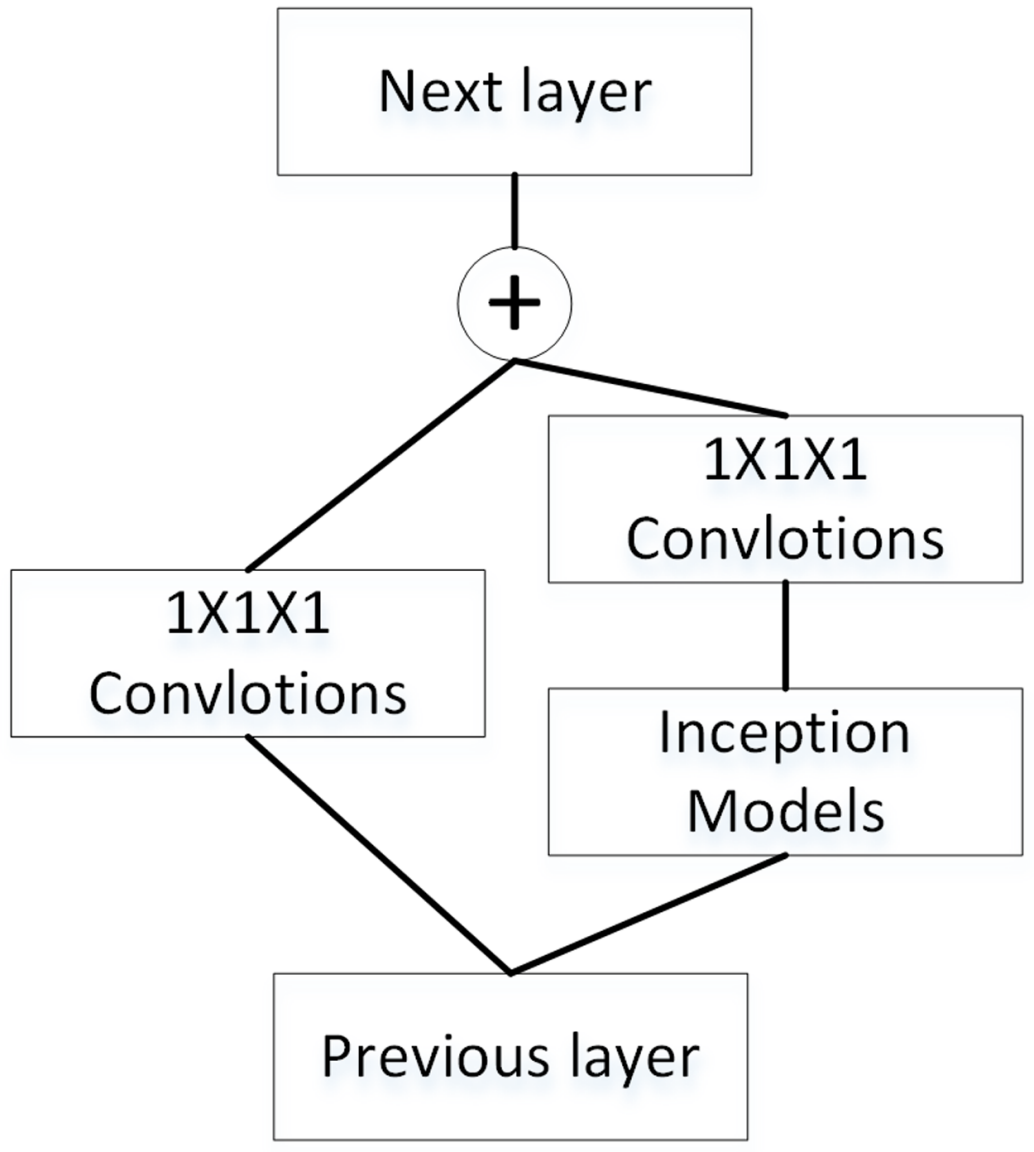
The overall architecture of the Inception-ResNet network.

The residual network was proposed to solve the problem of degradation in the deep neural network [19]. The assumption is that it should be easier to optimize the residual mapping than to optimize the original, unreferenced mapping. Inspired by Szegedy et al. [16], we also used the Inception-ResNet to classify the lung nodules in LIDC. The network structure is shown in Fig 6. Note that the Inception module in the Inception-ResNet is the same as Inception1 in this paper.

### Performance evaluation

For the binary classification, performance is quantitatively determined via sensitivity, specificity and error rate. The sensitivity measures the proportion of the positive samples that are correctly classified, the specificity measures the proportion of negative samples being correctly classified, and the error rate measures the proportion of samples that are misclassified. They are calculated using the true positive (TP), true negative (TN), false negative (FN), and false positive (FP) as follows:

$$Sensitivity=\frac{TP}{TP+FN}$$ (6)

$$Specificity=\frac{TN}{TN+FP}$$ (7)

$$Error\;\;\;\;\;rate=\frac{FP+FN}{TP+TN+FP+FN}$$ (8)

where TP is the number of positive examples classified as positive, FP is the number of negative examples classified as positive, TN is the number of negative examples classified as negative and FN is the number of positive examples classified as negative.

The Area Under the Curve (AUC) is shown when we compare the binary classification performance with that of other works. This curve refers to the Receiver Operating Characteristic (ROC) curve, a standard technique for summarizing classifier performance over a range of tradeoffs between true positive and false positive error rates. The AUC is an accepted traditional performance metric for an ROC curve and is equivalent to the probability that the classifier or feature will rank a randomly chosen positive instance higher than a randomly chosen negative instance. That is, the AUC can be used to represent the classifier's ability to distinguish samples.

## Experiments and results

### Setup

#### 3D volume data generation

**Fig 7:**
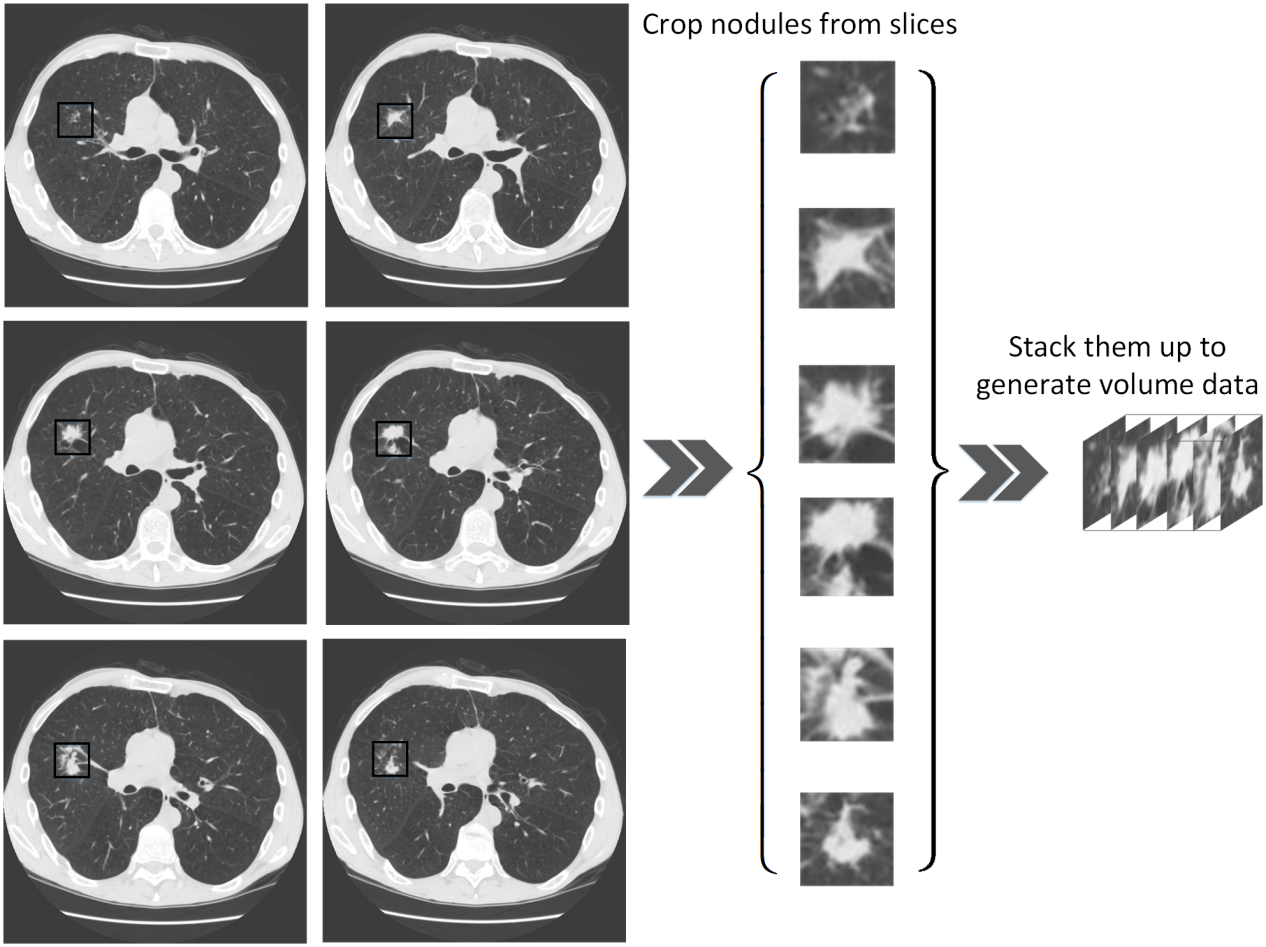
The process of generating 3D volume data.

In order to generate a 3D lung volume data set, we first established a 2D data set as was done in paper [31]. We grouped all slices at the nodule level, that is, all the slices belonging to the same nodule were assigned to the same group. In total, we found 776 nodules. 3D volume data were generated as shown in Fig 7. However, the number of slices belonging to the same nodule was different. In order to unify the number of slices of a nodule, we adopted a balanced strategy. Specially, for nodules with *n* slices, we choose slice 1, slice *n* and slice $1+\left[\frac{n-1}{5}\right]\cdot k$, where *k* = {1, 2, 3, 4} and [•] denotes the function rounding to an integer. For example, for a nodule with 10 slices, we chose slice 1, slice 3, slice 5, slice 6, slice 8 and slice 10. For a nodule with fewer than 6 slices, we used all-zero slices to fill them up to 6 slices.

#### Labeling

We carried out both binary and ternary classifications in our experiments. For the binary classification, we labeled lung nodules as benign if the diagnosis result was 1 at the nodule level, and malignant if the diagnosis result was 2 or 3 at the nodule level. If the diagnosis result at the nodule level was 0, then we labeled it in accordance with the diagnosis result at the patient level. However, in this paper, all slices belonging to the same nodule were considered to belong to the same sample. Ultimately, we found 29 benign patient cases and 67 malignant patient cases, with a total of 186 benign lesions and 590 malignant lesions. We augmented the data by performing rotations on each lesion. More specifically, each benign lesion was rotated by 9 degree intervals from 0 to 360 degrees, resulting in 7,440 benign lesions. In order to obtain a balanced dataset, we rotated each malignant lesion by 30 degree intervals from 0 to 360 degrees, eventually resulting in 7,080 malignant lesions.

As for the ternary classification, we labeled the extracted lesions as benign, malignant primary or malignant metastatic. Benign lesions were identified in the same way as those in the binary classification, while the malignant lesions were subdivided into primary malignant (if the diagnosis result was 2) and metastatic malignant (if the diagnosis result was 3). Ultimately, we found 29 benign patient cases, 25 primary malignant patient cases and 42 metastatic malignant patient cases, with a total of 186 benign lesions, 169 primary malignant lesions and 421 metastatic malignant lesions. Each benign lesion was rotated by 20 degree intervals from 0 to 360 degrees, resulting in 3,348 benign lesions. In order to obtain a balanced dataset, each primary malignant lesion was rotated by 18 degree intervals from 0 to 360 degrees, and each metastatic malignant lesions was rotated by 45 degrees intervals from 0 to 360 degrees, resulting in 3,380 primary malignant lesions and 3,368 metastatic malignant lesions. The number of patients and lesions are listed in Table 2.

**Table 2:** The number of patients and lesions.

	Class	The number of Patients	The number of lesions before rotating	The number of lesions after rotating
The binary classification	Benign	29	186	7,440
	Malignant	67	590	7,080
The ternary classification	Benign	29	186	3,348
	Malignant primary	25	169	3,380
	Malignant metastatic	42	421	3,368

#### Hyperparameters setting

The results in paper [31] showed that the BN layer did not reduce the error rate for 2D data in the LIDC dataset. So, in our experiment, we did not employ the BN layer throughout. Instead, it was replaced by the dropout layer. We set each dropout rate to be 0.5. We initialized the learning process with a learning rate of 0.001 and completed the learning process in 50 epochs using a batch-size of 16. The momentum was fixed to 0.9 with weight decay parameters set to 5×10^−4^ throughout the learning process. We used max-pooling in the chain architectures and all pooling sizes and pooling strides in them are set to 2×2×2, while they are set to 2×3×3 in the network with DAG architecture. A detailed explanation about the pooling size in the DAG architectures are shown in the discussion section. All convolutional strides were set to 1.

The performance of the binary classification was quantitatively determined via the error rate, sensitivity and specificity, while the performance of the ternary classification was measured only by the error rate, as sensitivity and specificity could only be defined in the binary classification. They were all obtained by 10-fold cross validation. Specifically, all samples were randomly divided into 10 groups. Then, 9 of them were selected as the training set, while the remaining group acted as the validation set. After each random grouping, 10 results could be obtained. It should be noted that there are some duplicate samples for each patient. Therefore, in order to ensure that every sample in the validation set is not the same as that in the training set, we divided the data at the patient level. In detail, the 96 patients were randomly divided into 10 groups, of which there are 6 groups with 10 patients and 4 groups with 9 patients. Therefore, the number of samples in each subset is different, but there is not much difference. We weighted (determined by the proportion of the number of samples in the validation set) the average of the 10 results to offer the final result for this random grouping. In order to reduce the randomness of the results, we ran a 10-fold cross validation 5 times for the same network, with the final results obtained by averaging the results.

### Training and the validation results

The experiment was divided into two parts. First, 3D MV-CNN with chain architecture, i.e., Softmax, CNN1, CNN2 and CNN3, was studied. The single-view strategy and the multiple-view strategy were all investigated for each network. We then explored 3D MV-CNN with DAG architecture, namely Inception1, Inception2 and Inception-ResNet. For the completion of the experiment, we used the keras library [36].

**Fig 8:**
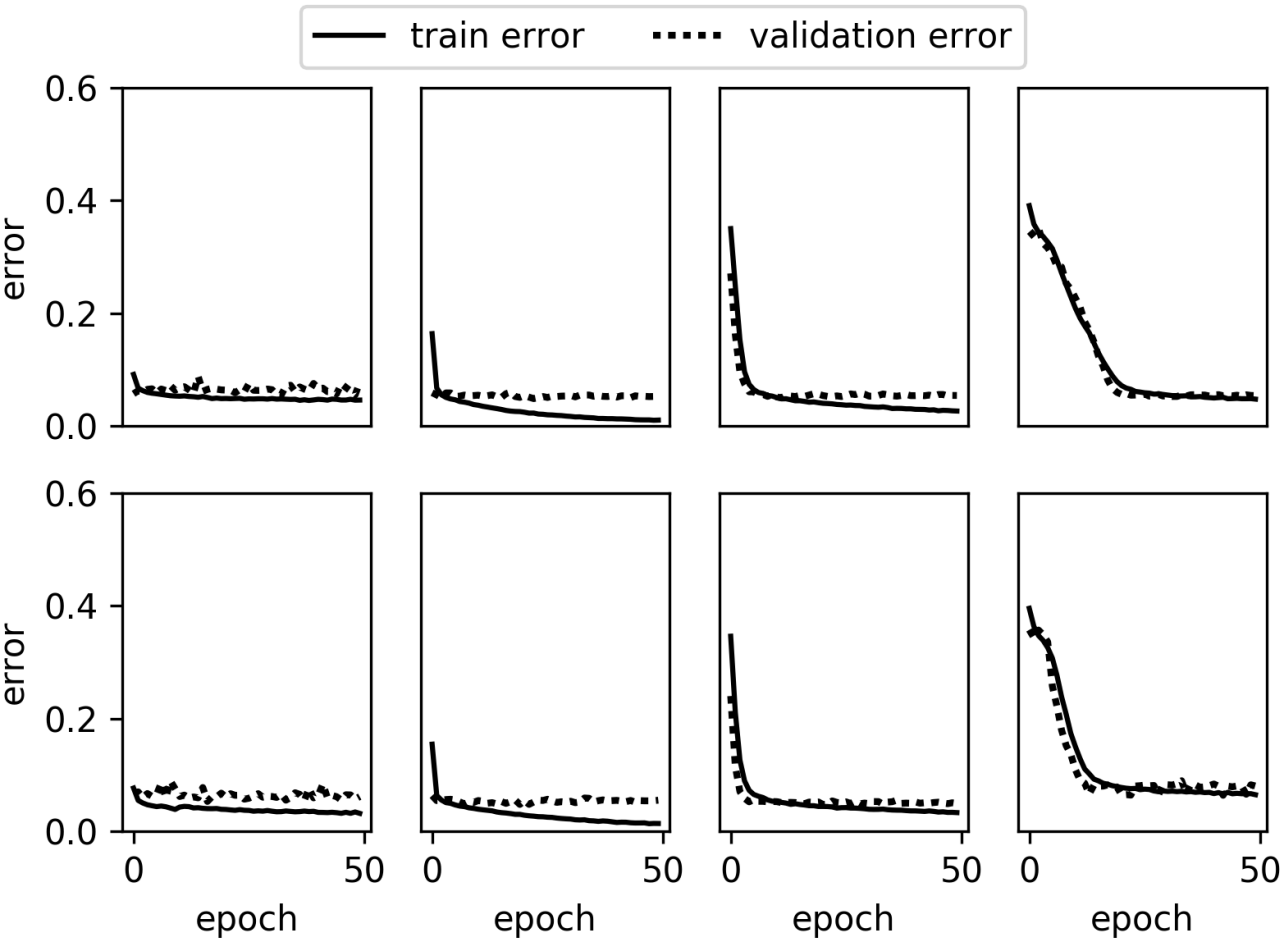
The error rate of the 3D SV-CNN (top) and 3D MV-CNN (bottom) with chain architecture for the binary classification. From left to right, there are the error rates of Softmax, CNN1, CNN2 and CNN3, respectively.

**Fig 9:**
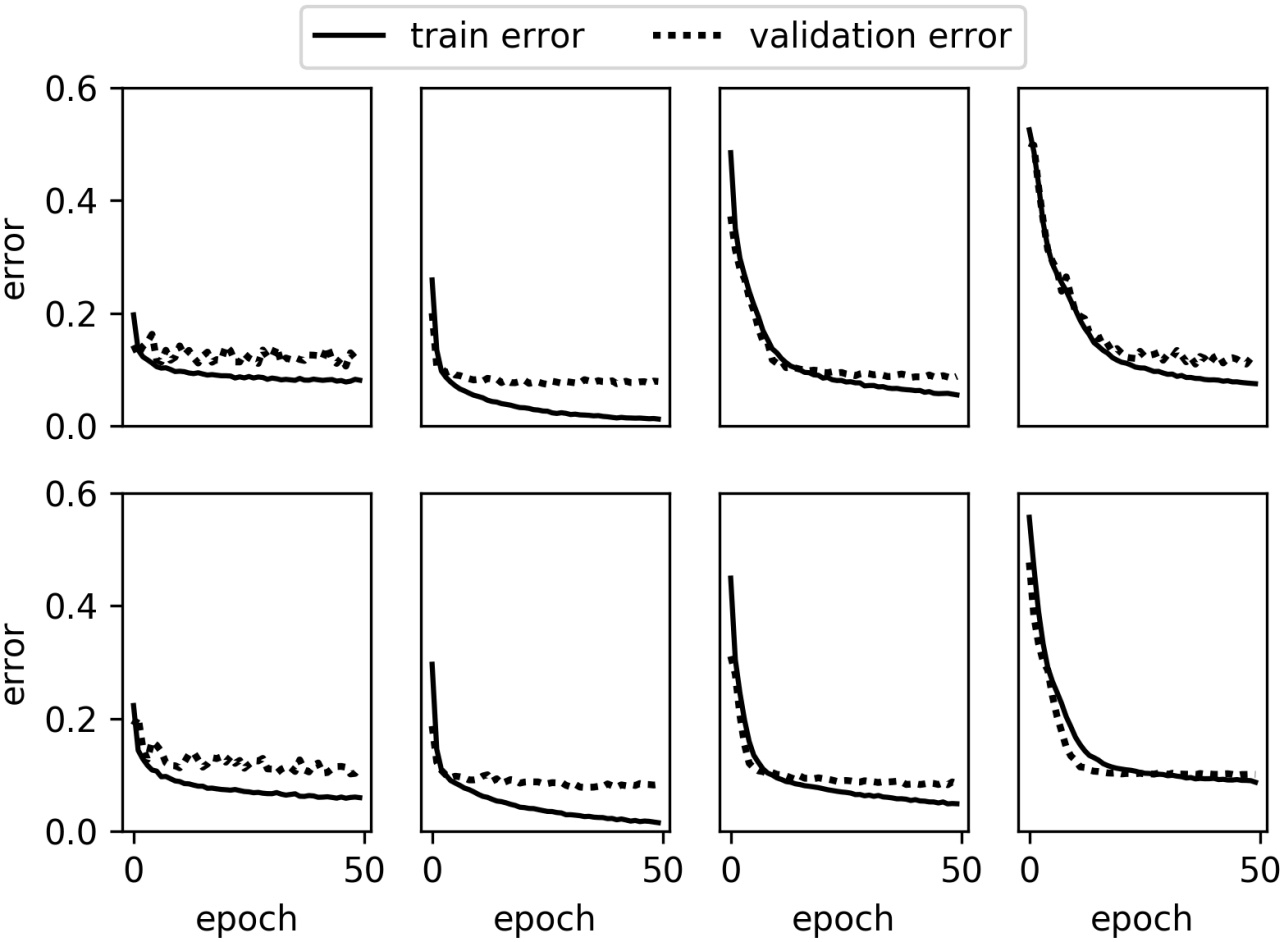
The error rate of the 3D SV-CNN (top) and 3D MV-CNN (bottom) with chain architecture for the ternary classification. From left to right, there are the error rates of Softmax, CNN1, CNN2 and CNN3, respectively.

The training curves of 3D CNN with chain architecture are shown in Figs 8 and 9. We noticed that, as the depth of the model increases, the model's error rate for the first epoch increases and more epochs are needed for the model to converge. This is because parameters in the layer being farther away from the loss layer are more difficult to optimize.

The validation error rate of 3D CNN with chain architecture is given in Tables 3 and 4. The corresponding results for 2D CNN in paper [31] are also shown. As can be seen from the table, the validation error rate of 3D CNN is generally lower than that of 2D CNN. Furthermore, for the ternary classification, it is CNN1 that obtains the lowest error rate for 3D CNN, while it is CNN3 for 2D CNN in paper [31]. This indicates that, for 3D CT lung imaging, the feature extraction capability of 3D convolution is better than that of 2D convolution. It is also worth noticing that the error rate of the multi-view network is generally lower than that of the single-view network. Therefore, the multi-view strategy for 2D CNN can also be used to improve the performance of the 3D CNN.

**Table 3:** The result of the binary classification for the networks with chain architecture.

The number of input channels	Network	2D CNN [31]			3D CNN		
		Error rate	Sensitivity	Specificity	Error rate	Sensitivity	Specificity
1	Softmax	6.49%	89.7%	97.22%	5.46%	94.81%	92.96%
	CNN1	5.66%	88.88%	99.67%	4.82%	95.56%	93.01%
	CNN2	5.60%	88.93%	99.72%	4.97%	95.51%	92.99%
	CNN3	5.87%	89.4%	98.74%	5.19%	**98.45%**	89.76%
3	Softmax	6.28%	**90.18%**	97.17%	5.29%	94.89%	93.07%
	CNN1	**5.41**%	88.92%	**100%**	**4.75%**	95.60%	**93.94%**
	CNN2	5.54%	89%	99.76%	4.76%	95.61%	93.89%
	CNN3	5.6%	88.97%	99.68%	6.38%	94.17%	89.73%

**Table 4:** The result of the ternary classification for the networks with chain architecture.

The number of input channels	Network	2D CNN Error rate [31]	3D CNN Error rate
1	Softmax	19.18%	10.59%
	CNN1	17.66%	8.12%
	CNN2	16.27%	8.42%
	CNN3	15.21%	10.94%
3	Softmax	18.19%	10.03%
	CNN1	18.16%	**7.78%**
	CNN2	18.05%	8.25%
	CNN3	**13.19**%	9.31%

In our experiment, the error rate of CNN1 was lower than CNN2 and CNN3, which may be due to hyper-parameters. If you did a strict grid search (which is too time consuming), you may get a different result. But it will not change too much; that is, from our experiments, we can at least conclude that using a 3D CNN to classify lung nodules in CT does not need a very deep network.

Furthermore, inspired by GoogLeNet, we also explored the performance of 3D Inception and 3D Inception-ResNet on the LIDC dataset. Two architectures were adopted for 3D Inception networks, as described in Fig 5. We use only one Inception layer, as the CNN with one convolution layer performs with the lowest error rate, as shown in Tables 3 and 4. All the results for the network with DAG architecture are shown in Table 5. Note that all the networks in this table employ the multi-view strategy. The results show that Inception1 achieves better results than all 3D CNNs with chain architecture. The interesting thing is the fact that Inception2 did not obtain a lower error rate than Inception1. In fact, while the decomposition in Inception2 reached greater deeper depths with fewer parameters, such depths seem to have no positive effect on the classification of our data taken from the LIDC dataset.

**Table 5:** The validation error rate of the classification for the 3D multi-view networks with DAG architecture.

Network	Binary classification			Ternary classification error rate
	Error rate	Sensitivity	Specificity	
3D MV-CNN1	4.75%	95.60%	93.94%	7.78%
Inception1	**4.59%**	**95.68%**	**94.51%**	**7.70%**
Inception2	4.97%	95.48%	94.32%	8.43%
Inception-ResNet	4.89%	95.64%	94.37%	8.79%

Therefore, we preferred to use Inception1 module in our Inception-ResNet. We also scaled down the residuals before adding them to the previous layer activation seemed to stabilize the training [16]. In this paper, the scaling factors were set to be 0.1. However, results show that our Inception-ResNet did not achieve better results than Inception1. Indeed, the residual connection is proposed to solve the gradient degradation problem, which often appears in deep neural networks [19]. Results reveal that the classification for our data does not require too many layers, so the residual connection would be of little help. In fact, the results show that, for our networks and data, the residual connection makes the performance worse.

### Comparing the results with other works

The results from our MV-CNN are quite competitive for the LIDC-IDRI dataset, as shown in Table 6. It should also be noted that the augmentation factors for benign/malignant or benign/primary/metastasis lesions are different in our experiment, and we tested all the images, including augmented samples. In papers [21] and [31], researchers used similar methods, but some other researchers, such as in papers [7], [24] and [37], did not include the augmented samples in the test set. In addition, different papers use different samples and data dividing methods. All of these make it difficult to achieve a fair comparison. Although each models in Table 6 uses the LIDC-IDRI dataset, they don’t use the same parts. “Diagnosis data” means a paper used data that had a clear diagnostic result, while “All” means a paper used each instance in the dataset; but labelled each in a unique way. The data partitioning method in each paper was also shown in Table 6. To the best of our knowledge, we could not discover any other works that involved ternary classification of the LIDC-IDRI database except for paper [31].

**Table 6:** Some classification results on LIDC-IDRI dataset.

Related work	Data sources	Data partitioning methods	Binary classification		Ternary classification error rate
			Error rate	AUC	
Han et al. [7]	All	50% training and 50% testing	----	0.93	----
Shewaye et al. [37]	Diagnosis data	65% for training and 35% for testing	16%	0.94	----
Kumar et al. [21]	Diagnosis data	80% for training, 10% for validation and 10% for testing	22.48%	----	----
Shen et al. [24]	All	5-fold cross validation	12.86%	0.93	----
Liu and Kang, [31]	Diagnosis data	10-fold cross validation	5.41%	0.98	13.19%
Our work	Diagnosis data	10-fold cross validation	**4.59%**	**0.99**	**7.70%**

**Fig 10:**
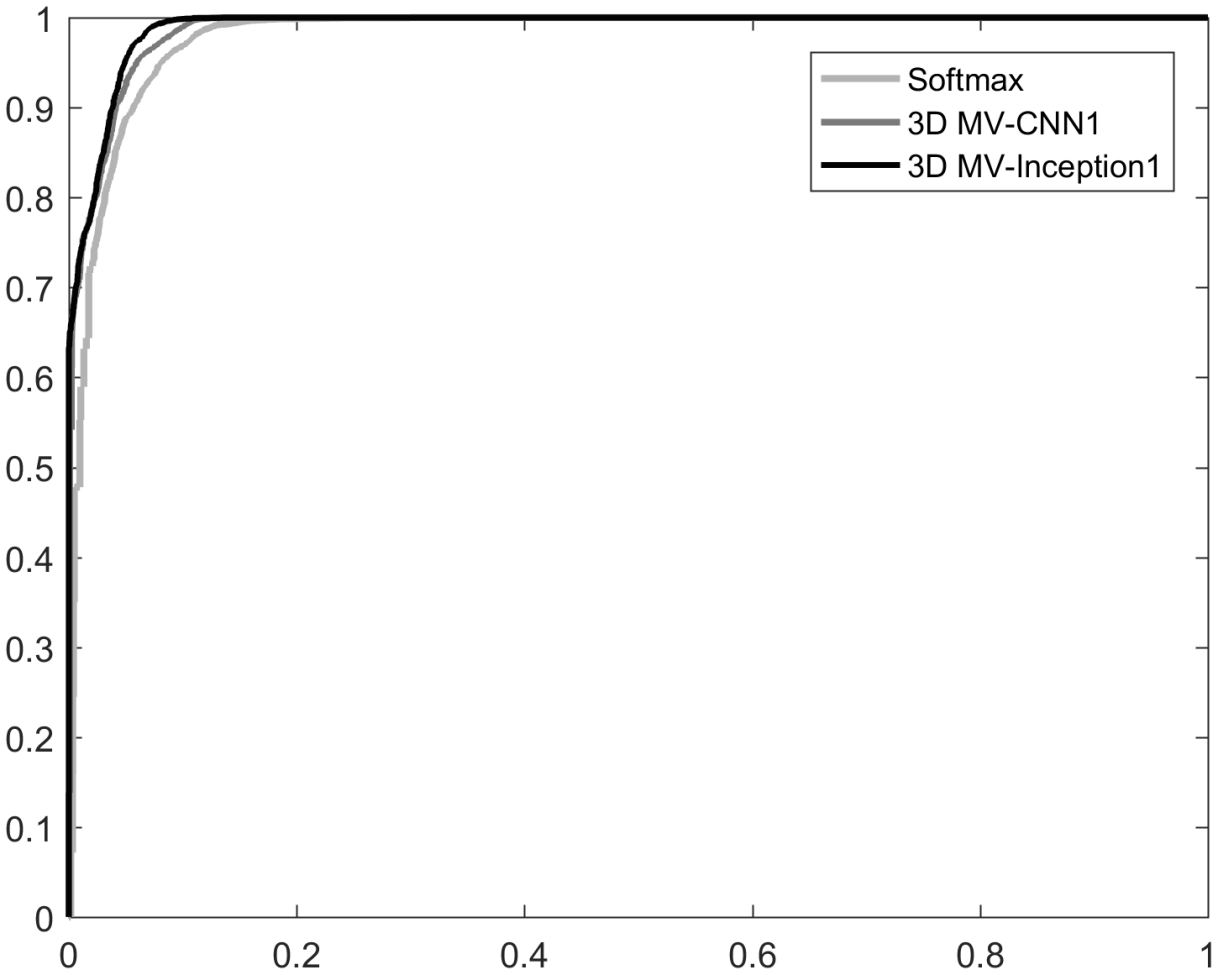
The ROC curves of MV-softmax, 3D MV-CNN1 and 3D MV-inception1.

The ROC curves of MV-softmax, 3D MV-CNN1 and 3D MV-inception1 are shown in Fig 10. The AUC of Softmax, 3D MV-CNN1 and 3D MV-Inception1 are 0.96,0.98 and 0.99, respectively. It can be seen that the results of all models are not bad, and that the advantage of 3D inception1 is small compared to the other two models, so that the curves are close to each other (which is why only three models of the roc curve are shown; otherwise, many curves would be mixed together so that it would be difficult to distinguish). This reveals that those samples, when correctly classified, are easy to distinguish, while the misclassified samples are so similar to the other category that it is difficult to make the correct classification. A better-performing model can only make corrections to a small portion of the misclassified samples.

## Discussion

### The number of parameters *vs*. training time and validation error rate

**Fig 11:**
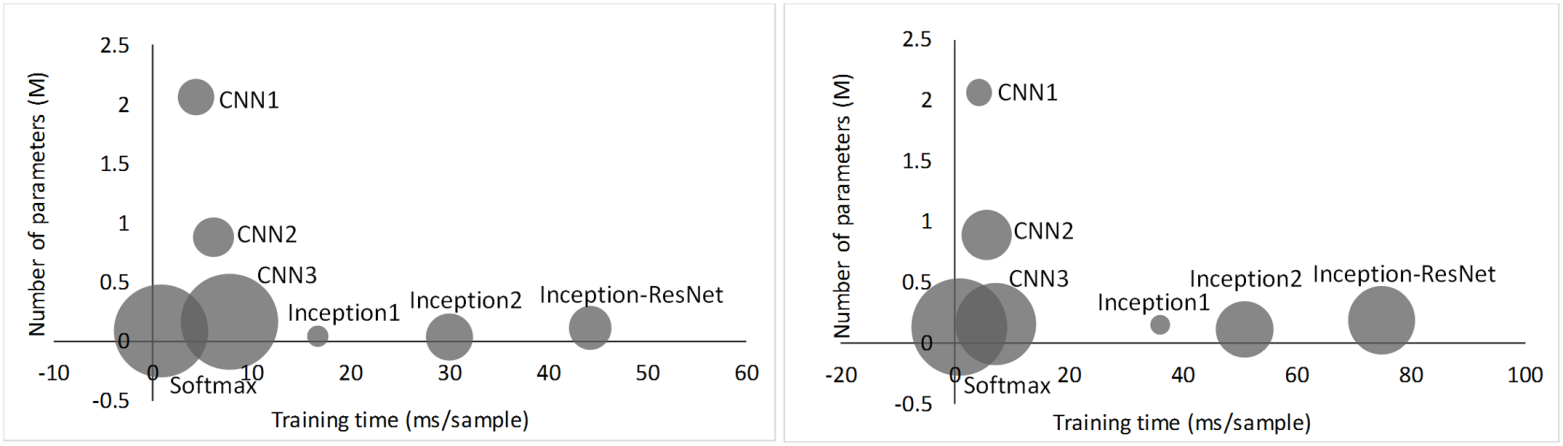
The number of parameters vs. training time and validation error rate for the binary classification (left) and the ternary classification (right). The area of the circles represents the corresponding validation error rate.

We compared the number of parameters, training time, and validation error rates for each model in this paper, as shown in Fig 11. All experiments were conducted on an NVIDIA GeForce GTX Taitan X. In these networks, Softmax and CNN3 are closest to the lower left corner. However, their error rates are higher than those of other networks. The training time of networks with DAG architecture is longer than that of networks with chain architecture, but their parameters are far fewer. This shows that the training time is not causally connected to the number of parameters. Note that DAG architecture is more complex than chain architecture. Therefore, as Fig 11 shows, the training time is deeply related to the complexity of the model architecture. Of all the networks, the Inception1 network achieved the lowest error rate. Although its training time was higher than the model with chain architecture, it was lower than Inception2 and Inception-ResNet. Furthermore, Inception1 has fewer parameters than those networks with chain architecture.

### Average pooling

**Fig 12:**
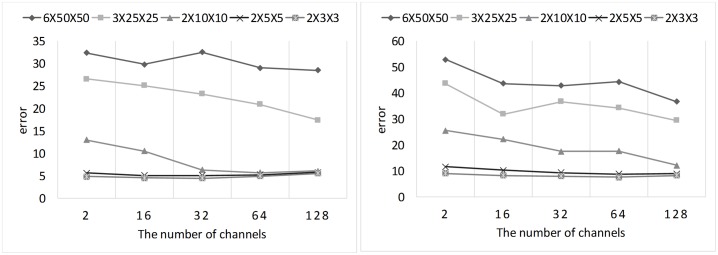
The result of grid search for the binary (left) and ternary (right) classification.

In this subsection, we explained why we did not fully follow the global average pooling method. We made a coarse grid search for the number of output channels in the last 1×1×1 convolutional layer and the average pooling size (a detailed grid search is time consuming, and it takes more than five hours for a cross validation). The results of grid search for the binary and ternary classification are shown in Fig 12.

If we used global average pooling, the number of channels in the last 1×1×1 convolutional layer would be 2 (for binary classification) or 3 (for ternary classification) and the average pooling size should be 6×50×50. However, the results show that the effect of this setting is very bad. As shown in Fig 12, for binary classification, when the number of channels is 32 and pooling size is 2×3×3, the model achieves its lowest error rate. For ternary classification, when the number of channels is 64 and the polling size is 2×3×3, the model achieves its best performance. Therefore, we used this setting in our experiment.

Global average pooling in Network-in-Network [14] and GoogLeNet [8,15,16] get good results because they have 1000 categories, so they have 1000 channels before the softmax layer. However, in our task, the number of categories was only 2 or 3. If we follow the global average pooling setting fully, the softmax classifier in the network has only 4 or 9 parameters, which is so few that it seriously affects the performance of the model. Increasing the number of channels and reducing the average pooling size appropriately will increase the number of parameters in the softmax classifier, which will improve the performance of the model. Of course, the number of these parameters cannot be too large, so we chose a setting that minimizes the the number of parameters without degrading the model performance.

### Compare with the one-view-one-network strategy

It should be noted that the Inception1 network in Table 5 use the multi-view-one-network strategy (as shown in Fig 2 bottom). In this section, we compare it to the one-view-one-network strategy (as shown in Fig 2 top), and the results are shown in Table 7. Two variants of the one-view-one-network strategy were tested. In the first variant (one-view-one-network1), the hidden layer of each sub-network had the same structure as the Inception1 network in Table 5. That’s why the number of parameters of one-view-one-network1 is almost 3 times that of the multi-view-one-network. In the second variant (one-view-one-network2), the number of channels in each layer was reduced so that the total number of parameters would almost equal that of the multi-view-one-network.

**Table 7:** Compare the multi-view-one-network strategy with the one-view-one-network strategy.

Strategy		Error rate	The number of parameters	Inference time (ms/sample)	*R*_*p*_	*R*_*f*_
Binary classification	One-view-one-network1	5.28%	1.40×10^5^	22.73	1:25	1:1
	One-view-one-network2	5.61%	0.46×10^5^	6.89	1:60	1:1
	Multi-view-one-network	**4.59%**	0.47×10^5^	10.17	1:25	3:1
Ternary classification	One-view-one-network1	8.13%	4.49×10^5^	46.06	1:33	1:1
	One-view-one-network2	9.07%	1.45×10^5^	10.90	1:80	1:1
	Multi-view-one-network	**7.70%**	1.49×10^5^	16.84	1:33	3:1

In Table 7, we can see that the multi-view-one-network strategy got a better result than the one-view-one-network strategy. Compared one-view-one-network2 with multi-view-one-network, one-view-one-network2 had fewer parameters, but got a higher error rate. Therefore, fewer parameters have nothing to do with the model getting a lower error rate.

We examined two ratios: *R*_*p*_ and *R*_*f*_ in Table 7, where *R*_*p*_ denotes the number of parameters in the convolutional layers compared to the number of parameters in the softmax layers (excluding the bios), and *R*_*f*_ denotes the ratio of the number of filters to the number of hidden channels. The values of *R*_*f*_ reveal that the multi-view-one-network strategy provides more filters when the number of hidden layers is constant. These filters make more connections between the input channel and the hidden layer channel. And each feature map in the hidden layer incorporates the information of all views. That’s why the inferring time of multi-view-one-network is more than that of one-view-one-network2. Because the parameters in the convolutional layer account for only a small part of the parameters of the entire network (because all the values of *R*_*p*_ are very small), the increase in the number of filters does not make the parameters of the model increase significantly.

## Conclusions

In this paper, 3D MV-CNN with the multi-view-one-network strategy is studied for its potential in the classification of lung nodules. We conducted two classification tasks: 1.) the binary classification, in which nodules are divided into benign and malignant, and 2.) the ternary classification, in which nodules are divided into benign, primary malignant and metastatic malignant. We explored the network with chain architecture and with a DAG architecture. For the CNN with chain architecture, the results showed that the multi-view-one-network strategy can aid in improving the classification performance of 3D CNN, and that 3D MV-CNN’s performance surpasses that of 2D MV-CNN by a significant margin. For 3D CNN with DAG architecture, all the networks employed the multi-view-one-network strategy. The 3D variants of Inception1 obtained the lowest error rate among all of the networks, with an error rate of 4.59% for the binary classification and 7.70% for the ternary classifications, better than other works on the classification of the LIDC dataset. The results show that, in addition to improving accuracy, the 3D Inception1 had fewer parameters than 3D MV-CNN with chain architecture, due to the fact that we use the 1×1×1 convolutional layer and the average pooling to replace the fully connected layer. We compare the multi-view-one-network strategy with the one-view-one-network strategy. The results reveal that the multi-view-one-network strategy can achieve a lower error rate than the one-view-one-network strategy. In the future, we aim to investigate the effect of this model on the automatic detection of lung nodules in CT combined with object detection techniques.
